# Bilateral Adrenal Tumors: A Visual Case Series

**DOI:** 10.1016/j.aace.2024.11.006

**Published:** 2024-11-27

**Authors:** Dipika R. Mohan, Rutu Shah, Malak Itani, Mohamed Awali, Sina Jasim

**Affiliations:** 1 Department of Medicine, School of Medicine, Washington University in St. Louis, St. Louis, Missouri; 2 Division of Endocrinology, Metabolism and Lipid Research, School of Medicine, Washington University in St. Louis, St. Louis, Missouri; 3 Mallinckrodt Institute of Radiology, Washington University in St. Louis, St. Louis, Missouri

**Keywords:** adrenal, adenoma, pheochromocytoma, imaging

## Introduction

Incidental adrenal masses are common, affecting > 6% of those above 60 years of age and are commonly unilateral and hormonally silent. The incidence of these tumors has risen sharply in recent years, and a minority of patients may have incidentally discovered bilateral masses.^1^^,^^2^ The vast majority of adrenal incidentalomas are benign, nonfunctional tumors of adrenocortical origin; however, these also include hormone-secreting adrenocortical adenomas, adrenocortical carcinomas (ACCs), myelolipomas, pheochromocytomas, adrenal lymphomas, and metastatic disease.

Simultaneous bilateral adrenal masses are significantly less common, and the differential diagnosis for these masses diverges from unilateral masses. Bilateral adrenal masses are more frequently associated with malignancy and tumor susceptibility syndromes.^2^ They are also more likely to be associated with hormonal disturbances such as mild autonomous cortisol secretion.^2^ Mild autonomous cortisol secretion confers a higher risk of cardiovascular and metabolic comorbidities, in some cases warranting surgical intervention.

## Case 1: Bilateral Adrenal Adenomas

**Figure 1:**
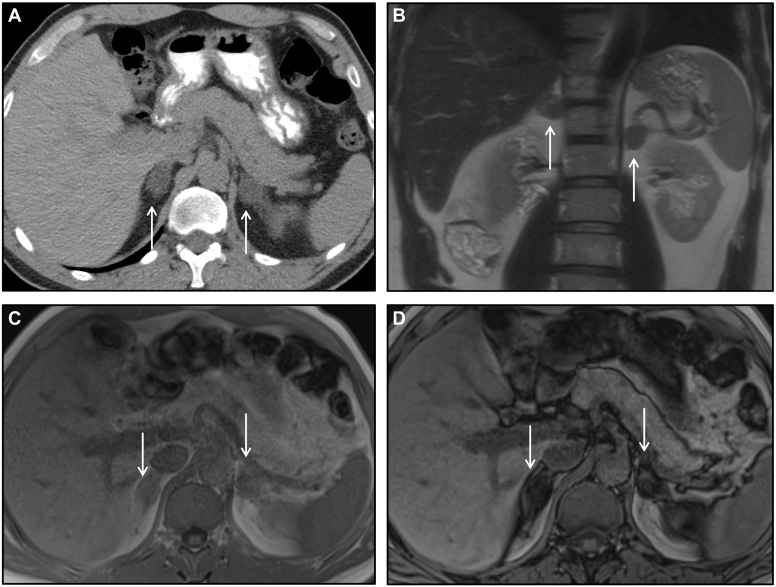
Bilateral adrenal adenomas. (*A*) Axial noncontrast CT image demonstrates bilateral adrenal nodules (arrows) with homogenous low density <10 HU, highly specific for benign lipid-rich adenoma. (*B*) Axial T2 -weighted image in the same patient shows homogenous low-signal intensity signal of the adrenal lesions (arrows) comparable to the liver. (*C*, *D*) Chemical shift MRI images (C in-phase, D opposed-phase) showing signal drop on opposed-phase images (arrows in D) where the lesions are now much darker than in C, consistent with intralesional fat, classic of lipid-rich adenomas. *CT* = computed tomography; *MRI* = magnetic resonance imaging.

A 55-year-old male presented with history of tobacco use disorder. Bilateral adrenal masses were incidentally discovered on screening computed tomography (CT) chest. His past medical history is notable for germline *BRCA1* mutation and occupational exposure to carcinogens (artificial foam fluid). Imaging characteristics favor these masses to be benign, lipid rich adenomas (Fig. 1). Patient has no signs or symptoms of hormone excess, corroborated by wholly negative biochemical evaluation. These masses have remained stable for several years.

## Case 2: Bilateral Macronodular Adrenal Hyperplasia in McCune-Albright Syndrome

McCune-Albright Syndrome (MAS) is a rare genetic disorder caused by somatic, postzygotic activating mutations in *GNAS*, a component of the protein kinase A (PKA) signaling pathway. Patients with MAS present with a variety of syndromic features resulting from constitutive PKA signaling across tissues. PKA signaling is critical for cortisol production; patients with MAS develop autonomous cortisol production via bilateral macronodular adrenal hyperplasia.^1^

**Figure 2:**
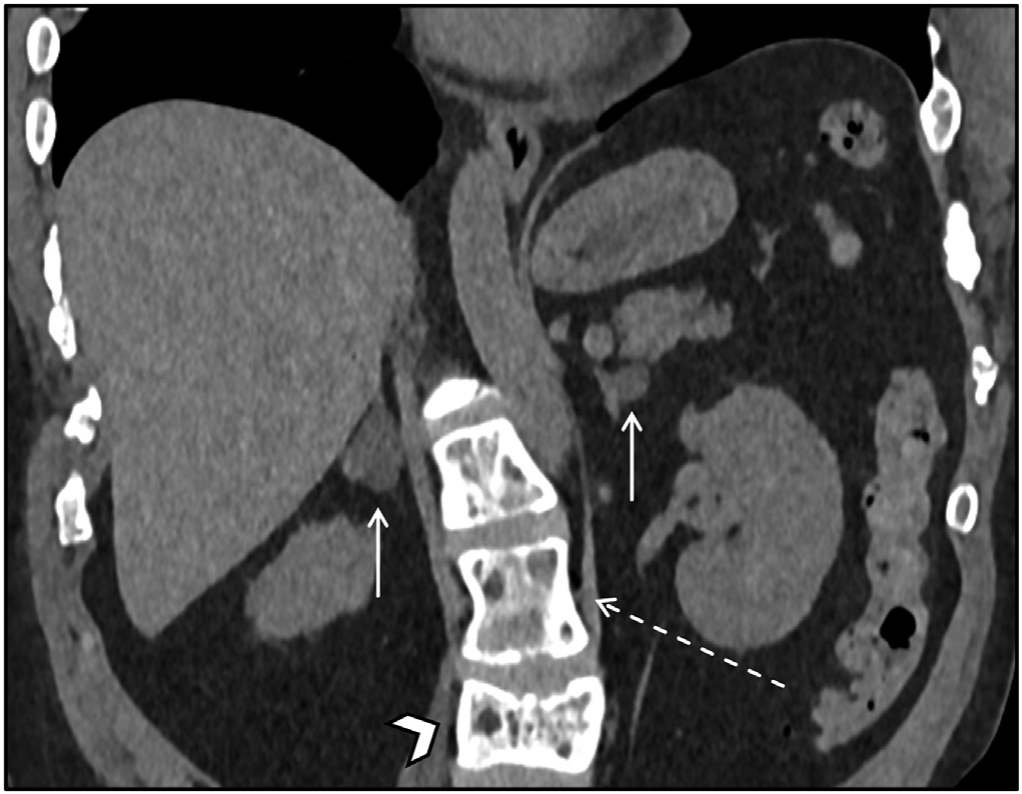
Bilateral macronodular adrenal hyperplasia in McCune-Albright Syndrome. Coronal noncontrast CT image demonstrates diffuse nodular enlargement of the adrenal glands bilaterally with homogeneous low-density lesions (arrows) consistent with bilateral nodular adenomatous hyperplasia in the setting of McCune-Albright Syndrome. Note diffuse bone abnormalities in keeping with multiple areas of fibrous dysplasia, with bone expansion and ground glass appearance (dashed arrow), variable rib spacing, and a vertebral compression deformity (arrowhead). *CT* = computed tomography.

Exemplifying this condition, we present a 38-year-old female with MAS, clinically manifested by precocious puberty, renal phosphate wasting with osteomalacia, fibrous dysplasia of bone with history of multiple fractures, growth hormone excess, and multinodular thyroid goiter requiring total thyroidectomy. A surveillance CT abdomen showed bilateral macronodular adrenal hyperplasia (Fig. 2).

Patient had unequivocal biochemical evidence of autonomous cortisol secretion. AM cortisol was 32.5 ug/dL (6.2-19.4 ug/dL) after low-dose dexamethasone suppression test (DST), and 27.3 ug/dL after high-dose DST. Late night salivary cortisol was 0.518 ug/dL (< 0.010-0.090 ug/dL). Total 24-hour urine cortisol was 78 ug (6-42 ug) then increased further on follow up. Adrenocorticotropic hormone (ACTH) was < 1.5 pg/mL (7.2-63.3 pg/mL).

Though she was initially asymptomatic, she later developed a number of symptoms attributed to hypercortisolism (easy bruising, recurrent skin infections, mood lability, and diverticulitis) which were refractory to medical management. She subsequently underwent bilateral adrenalectomy with symptom resolution.

## Case 3: Bilateral Pheochromocytomas in Von-Hippel Lindau Syndrome

Pheochromocytomas are catecholamine-producing tumors derived from the adrenal medulla. While these tumors can be sporadic, up to 40% occur as part of a tumor susceptibility syndrome.^3^ Clinical clues suggesting syndromic pheochromocytoma include bilaterality, the presence of contemporaneous or prior neuroendocrine tumors, and familial disease. Von-Hippel Lindau syndrome (VHL) is a tumor susceptibility syndrome caused by inactivating germline mutations in *VHL.* Patients with VHL are predisposed to a variety of tumors affecting neural, endocrine, and renal tissues that are exquisitely responsive to hypoxia-dependent signaling; they frequently develop pheochromocytomas.

**Figure 3:**
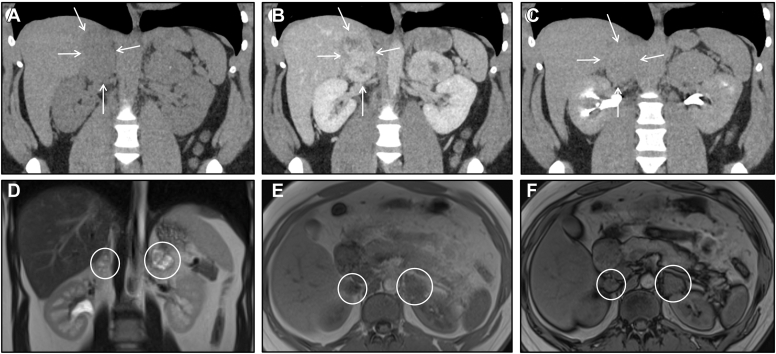
Bilateral pheochromocytomas in Von-Hippel Lindau syndrome. (*A*-*C*) Coronal adrenal-protocol CT before contrast (*A*), in the 75-second-delay phase (*B*), and in the 15-minute-delay phase (*C*) demonstrate bilateral heterogeneously hyperenhancing adrenal masses, *right* greater than *left* (white arrows) consistent with bilateral pheochromocytomas in the setting of Von-Hippel Lindau syndrome. (*D*-*F*) Coronal T2 image (*D*) demonstrate bilateral heterogeneously hyperintense T2 lesions, in keeping with classic light-bulb sign on T2 for pheochromocytomas (circles). Axial in-phase (*E*) and opposed-phase (*F*) images demonstrate no change in signal, in contrast to adenomas in Fig. 1. *CT* = computed tomography.

A 28-year-old female presented with a history of palpitations, anxiety, chest pain, diaphoresis, and gestational hypertension years before diagnosis. She developed abdominal pain and CT abdomen/pelvis demonstrated large (> 4 cm) bilateral adrenal nodules (Fig. 3 *A*–*C*); biochemical evaluation revealed elevated serum and total 24h urine metanephrines. These masses were hypermetabolic by fluorodeoxyglucose positron emission tomography (FDG-PET). She received alpha blockade and subsequent bilateral adrenalectomy. Pathology demonstrated that both masses were pheochromocytoma. Interestingly, the smaller right mass (6.8 cm in final specimen) had histological features concerning for aggressive behavior, including high proliferation rate with necrosis and vascular invasion; the larger left mass (8.3 cm in final specimen) had relatively benign histologic features. She was subsequently found to have well-differentiated neuroendocrine tumors of her gallbladder, which were resected. Germline testing revealed an inactivating mutation in *VHL*.

To illustrate the appearance of pheochromocytoma by magnetic resonance imaging (MRI), we also included images from a 20-year-old male patient with VHL and bilateral pheochromocytomas (Fig. 3 *D*–*F*).

## Case 4: Bilateral Adrenal Hemorrhage Due to Malignancy

Spontaneous bilateral adrenal hemorrhage is rare with a reported incidence of 0.14% to 1.8%.^4^ Most cases are associated with sepsis, anticoagulation, hematologic disorders, and trauma but in rare cases, can be seen in the setting of malignant adrenal tumors – primary or metastatic. Malignancies most commonly associated with adrenal metastases originate from lung, breast, gastrointestinal, and genitourinary tissue; melanoma may also metastasize to the adrenal glands.

**Figure 4:**
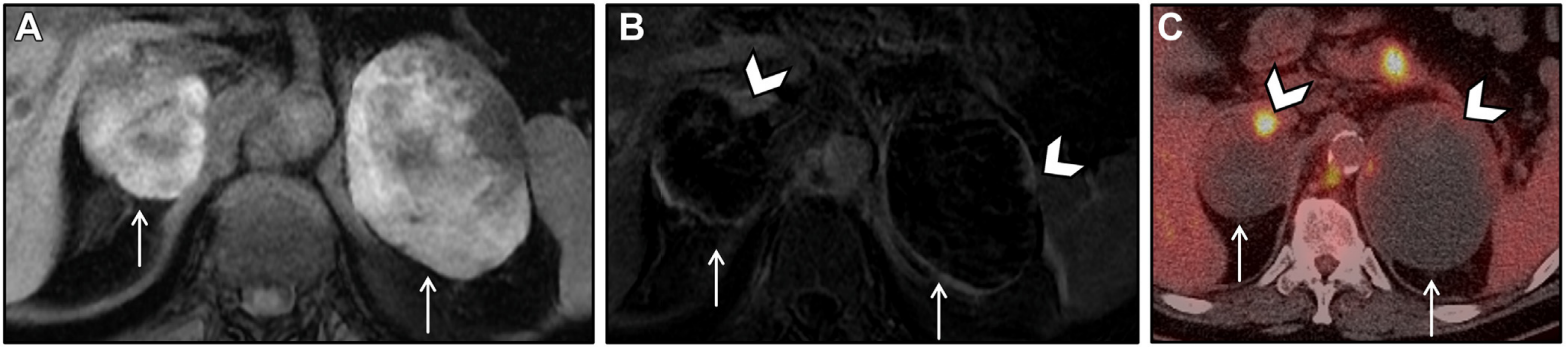
Bilateral adrenal hemorrhage due to malignancy. (*A*) Axial T1-weighted MR image showing bilateral adrenal lesions (arrows) with intrinsic T1 hyperintensity consistent with hemorrhagic lesions. (*B*) Axial subtracted MRI post contrast image showing only small areas of peripheral enhancement (arrowhead) with much of the lesion without enhancement. Findings consistent with bilateral metastatic disease with hemorrhage. (*C*) Axial fused FDG PET/CT image in the same patient showing the bilateral lesions with minimal focal peripheral increased FDG uptake correlating to the areas of enhancement seen on the MRI, related to viable tumor with adjacent hemorrhage. *CT* = computed tomography; *FDG PET* = fluorodeoxyglucose positron emission tomography; *MRI* = magnetic resonance imaging.

A 53-year-old male presented with a 4-week history of fatigue, emesis, and intermittent abdominal pain radiating to the back. CT and MRI scans initially demonstrated bilateral adrenal hemorrhage. On biochemical evaluation, he had a stimulated cortisol of 17.4mcg/dl (ref > 14 mcg/dl) on ACTH stimulation test, significantly elevated ACTH at 103.8 pg/nl (ref 7.0-63.0 pg/ml) and normal metanephrines, aldosterone, dehydroepiandrosterone sulfate, and electrolytes. He remained hemodynamically stable but was started on prednisone 5 mg daily due to high risk for primary adrenal insufficiency. Subsequent evaluation with FDG-PET demonstrated foci of FDG avidity in enlarging bilateral adrenal masses, a new FDG-avid periaortic retroperitoneal mass, and FDG-avid metastases to the pancreas. In retrospect, the largest focus of FDG avidity corresponded to a region of enhancement demonstrated on initial MRI (Fig. 4). Biopsy of the retroperitoneal mass revealed a high-grade sarcoma. Oncology started treatment with dexrozoxane and doxorubicin. His adrenal masses remained unchanged on follow-up CT 1 month later, and the patient passed away due to rapid progression of disease.

## Case 5: Bilateral Adrenal Myelolipomas in Congenital Adrenal Hyperplasia

Myelolipomas are hormonally inactive benign tumors composed of mature fat and hematopoietic elements that commonly arise from the adrenal cortex but can also be perinephric or presacral. Radiographically, they appear as round or ovoid well-circumscribed lesions with internal macroscopic fat content leading to low attenuation values between −30 and −100 HU. In the general population, the prevalence of myelolipomas is low at 0.4%, and myelolipomas tend to be unilateral with an average size of 2.3 cm.^5^ However, patients with congenital adrenal hyperplasia (CAH) have a much higher prevalence at 7%.^6^ In these patients, tumors are typically larger (average size 10 cm) and often bilateral. The exact mechanism is unknown; the prevailing hypothesis is that these tumors grow as a result of chronic ACTH stimulation.

**Figure 5:**
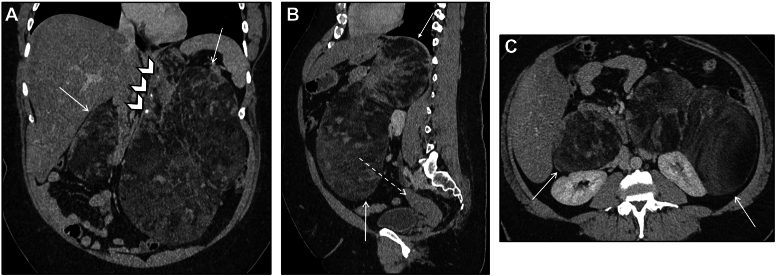
Bilateral adrenal myelolipomas in congenital adrenal hyperplasia. Coronal (*A*), sagittal (*B*), and axial (*C*) contrast-enhanced CT images on the 75 s delayed phase show bilateral large adrenal masses (arrows) with macroscopic fat and a few coarse calcifications (arrowheads) consistent with bilateral myelolipomas. Note this male patient has a uterus as they were assigned female at birth (dashed arrow). *CT* = computed tomography.

Here, we present a 37-year-old male, assigned female at birth, with a history of poorly controlled CAH who presented with left-sided abdominal pain, fatigue, and nausea. Although initially started on hormone replacement with prednisone and fludrocortisone at the time of diagnosis in early infancy, he was lost to follow up and had been off treatment for 20 years. Abdominal CT revealed large bilateral adrenal masses containing predominantly macroscopic fat. The right adrenal mass measured 21 cm and the left adrenal mass measured 33 cm (Fig. 5). Biochemical workup was notable for an elevated 17-hydroxyprogesterone of 40 746 (ref 42-196 mg/dl) and androstenedione/testosterone ratio of 1.7 (ref < 0.5 for adequate treatment), consistent with poorly controlled CAH. He restarted hormone therapy with significant improvement in symptoms and biochemical markers.

## Case 6: Bilateral Adrenocortical Neoplasms in Multiple Endocrine Neoplasia, Type 1

Multiple endocrine neoplasia type 1 (MEN1) is a genetic syndrome caused by inactivating germline mutations in the gene *MEN1*, encoding a chromatin scaffolding protein. Patients with MEN1 develop neuroendocrine tumors in a variety of tissues; these patients also have a higher risk of developing macronodular hyperplasia and ACC.^1^

**Figure 6:**
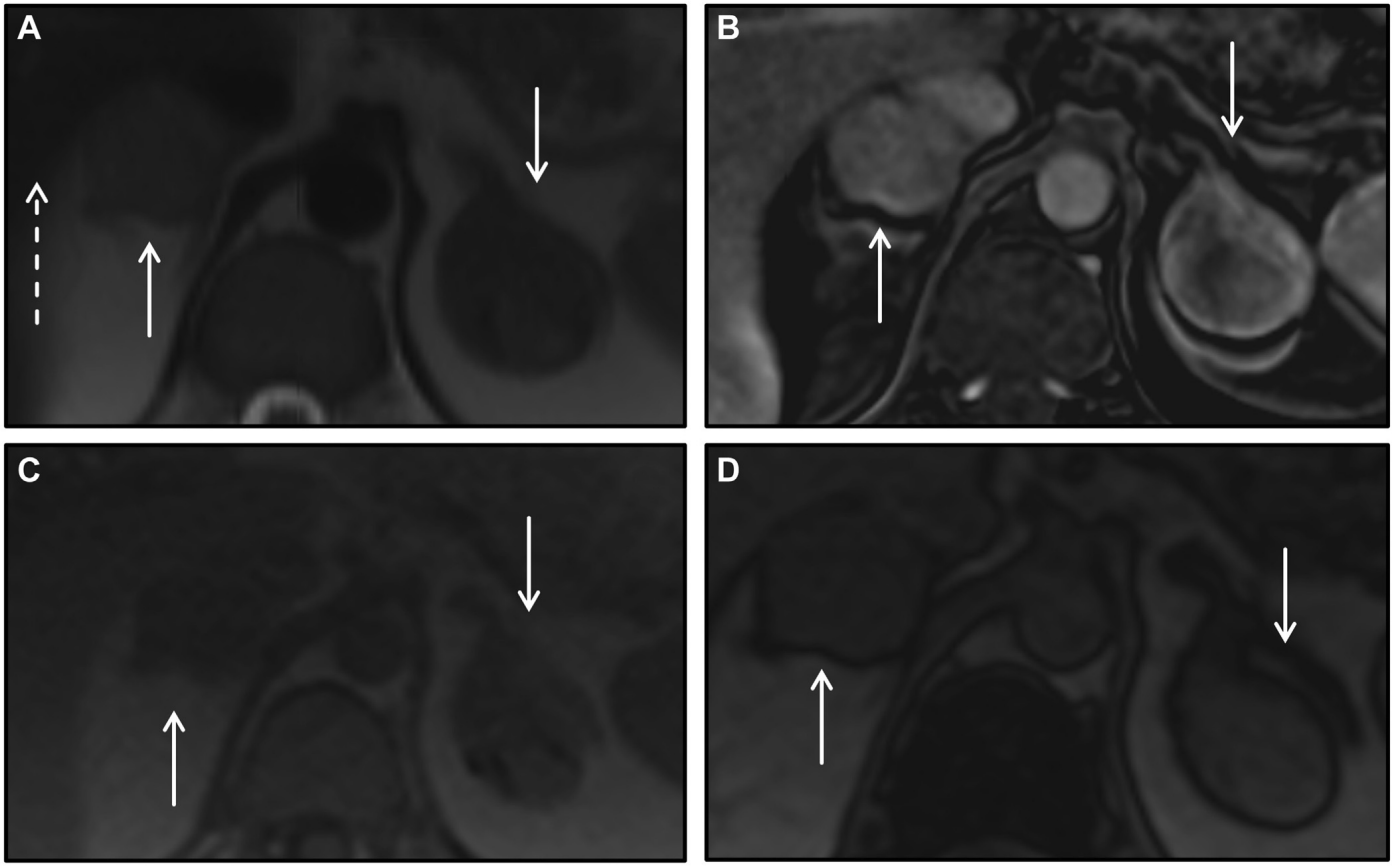
Bilateral adrenocortical neoplasms in multiple endocrine neoplasia type 1. (*A*) Axial T2 MRI image showing bilateral lesions with T2 intermediate signal, mildly above that of the adjacent liver (dashed arrow). (*B*) Axial post contrast image demonstrates heterogenous enhancement. (*C*-*D*) Axial in-phase (*C*) and opposed-phase (*D*) MRI images do not show any signal loss. Findings are not typical of adenomas. *CT* = computed tomography; *MRI* = magnetic resonance imaging.

Here, we present a 32-year-old female who first came to clinical attention due to infertility, found to have a prolactinoma. Genetic testing of the patient and first-degree relatives confirmed germline *MEN1* mutation. She eventually developed hyperparathyroidism, for which she received parathyroidectomy, and a pancreatic neuroendocrine neoplasm. She was also found to have bilateral adrenal masses – the left mass was large, > 4 cm, with complex features; the right mass was considered to be a smaller (< 4 cm) lipid-poor adenoma (Fig. 6). On subsequent imaging, patient’s right adrenal mass enlarged. She developed signs and symptoms of hypercortisolism (fatigue, weight gain, myalgias); adrenal venous sampling demonstrated cortisol production from both glands, right greater than left. She underwent right adrenalectomy which demonstrated an oncocytic adrenocortical neoplasm of unknown malignant potential.

Postoperative biochemical evaluation (inclusive of postoperative AM cortisol and DSTs) suggested persistent cortisol secretion. The patient subsequently developed recurrent symptoms of hypercortisolism and underwent left adrenalectomy 2 years later. On pathology, this lesion was found to be ACC with high risk of recurrence, and patient was started on adjuvant mitotane. Three years later, she developed a right-sided retrocaval nodule, which was initially concerning for recurrence. This was resected and found to be benign nodular adrenal hyperplasia. She remains ACC free on hormone supplementation.

## Case 7: Bilateral Adrenal Hemorrhage Due to Trauma

**Figure 7:**
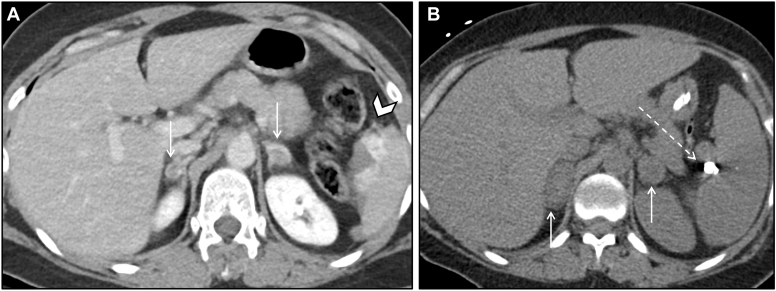
Bilateral adrenal hemorrhage due to trauma. Axial contrast-enhanced CT in the portal venous phase (*A*) demonstrates bilateral adrenal hyperattenuating lesions (arrows) that were new from a prior CT scan done 9 months ago. These enlarged on subsequent CT performed 12 days later (*B*), consistent with bilateral adrenal hemorrhage. Initial CT additionally shows active bleeding from a splenic laceration (arrowhead), with splenic artery embolization coils noted on subsequent CT (dashed arrow). *CT* = computed tomography.

In contrast to Case 4, here we present a more common cause of bilateral adrenal hemorrhage – trauma. This patient was a 50-year-old-female pedestrian struck by a car and pinned between the car and a concrete post. She had abdominal imaging a few months prior to the trauma with normal adrenal glands. At the time of the accident, she presented with numerous axial and appendicular fractures, in hemorrhagic shock caused by injury to multiple internal organs. In addition to hemodynamic support, she received urgent splenic artery embolization; she was found to have bilateral adrenal hemorrhage (Fig. 7).

## Case 8: Bilateral Adrenal Metastases From Melanoma

The vast majority of benign adrenal tumors are adrenocortical adenomas; in contrast, the most common malignant tumors of the adrenal gland are metastases.^2^ Most patients are asymptomatic but can have localized symptoms of back and abdominal pain due to mass effect or complications like local invasion or retroperitoneal hemorrhage (e.g. case 4). They can seldom result in primary adrenal insufficiency with significant involvement of bilateral adrenal tissue.^2^

**Figure 8:**
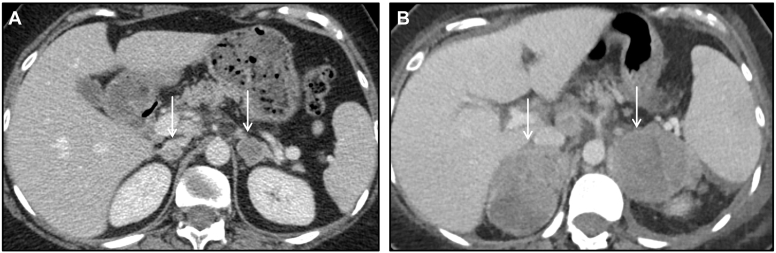
Bilateral adrenal metastases from melanoma. (*A*) Axial CT image with contrast showing bilateral adrenal lesions with heterogenous enhancement in a patient with known metastatic melanoma. (*B*) Axial CT with contrast 6 months later shows increase in size of the bilateral lesions, consistent with progressive metastatic disease. *CT* = computed tomography.

Here, we present a case of a 31-year-old female with melanoma on immunotherapy. She was found to have bilateral adrenal nodules on surveillance imaging. The original CT revealed 1.8 cm right and 2.2 cm left adrenal nodules. Follow-up CT scans at 6- and 9-month intervals demonstrated progressive bilateral enlargement to up to 7 cm on the right and 5.2 cm on the left, favoring metastatic disease (Fig. 8). Despite significant adrenal involvement, she had an intact hypothalamic-pituitary-adrenal axis. Specifically, she responded appropriately to ACTH stimulation test, with a base cortisol of 12.2 (ref 4.8-19.5mcg/dl) and stimulated cortisol of 22.0mcg/dl, ruling out adrenal insufficiency. She eventually passed away from complications of her advanced metastatic disease.

## Case 9: Bilateral Adrenocortical Neoplasms in Suspected Li-Fraumeni Syndrome

Li-Fraumeni syndrome (LFS) is a tumor susceptibility syndrome caused by inactivating germline mutations in *TP53*, encoding cell cycle and genome integrity regulator p53. Patients with LFS are vulnerable to a number of malignancies including sarcomas, hematologic malignancies, lung cancer, breast cancer, and both pediatric and adult ACC. Up to 5% of patients with LFS develop ACC. Nearly half of all ACC (including sporadic forms) possess genetic alterations targeting pathways governed by p53, and ACC harboring these mutations tend to secrete cortisol.^7^ As ACC is a rare malignancy with an annual incidence of 1 in a million, all patients with a new diagnosis of ACC should undergo germline testing for tumor susceptibility syndromes.

**Figure 9:**
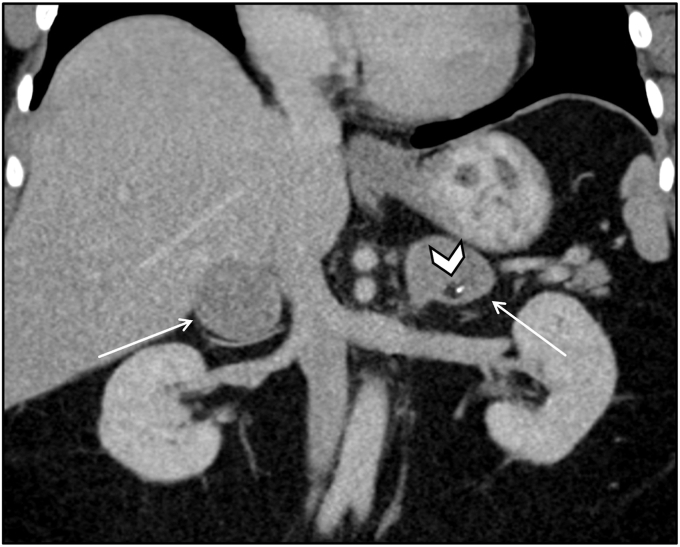
Bilateral adrenocortical neoplasms in suspected Li-Fraumeni syndrome. Coronal post contrast CT image showing bilateral adrenal lesions (arrows). The *right* adrenal lesion demonstrates heterogenous enhancement and was consistent with an adrenocortical carcinoma. The *left* adrenal lesion is heterogenous with foci of fat and calcifications (arrowhead) corresponding to sites of myelolipomatous change within this atypical adenoma. *CT* = computed tomography.

This is a 61 -year-old female with past medical history of postmenopausal bleeding and mesenteric fibrosis on adjuvant imatinib. She was initially found to have bilateral adrenal masses, which were initially stable on annual surveillance imaging, though the right adrenal mass slowly enlarged over the course of 6-7 years (Fig. 9). Hormonal work-up demonstrated combined cortisol and androgen (dehydroepiandrosterone sulfate) hypersecretion with ACTH suppression.

Patient underwent staged adrenalectomy with resection of right adrenal mass followed by resection of left adrenal mass months later. Right adrenal mass was found to be ACC with focal evidence of hemorrhage. Left adrenal mass was found to be an atypical adrenocortical adenoma with hemorrhage, myelolipomatous, and metaplastic changes.

## Case 10: Bilateral Adrenal Lymphoma

**Figure 10:**
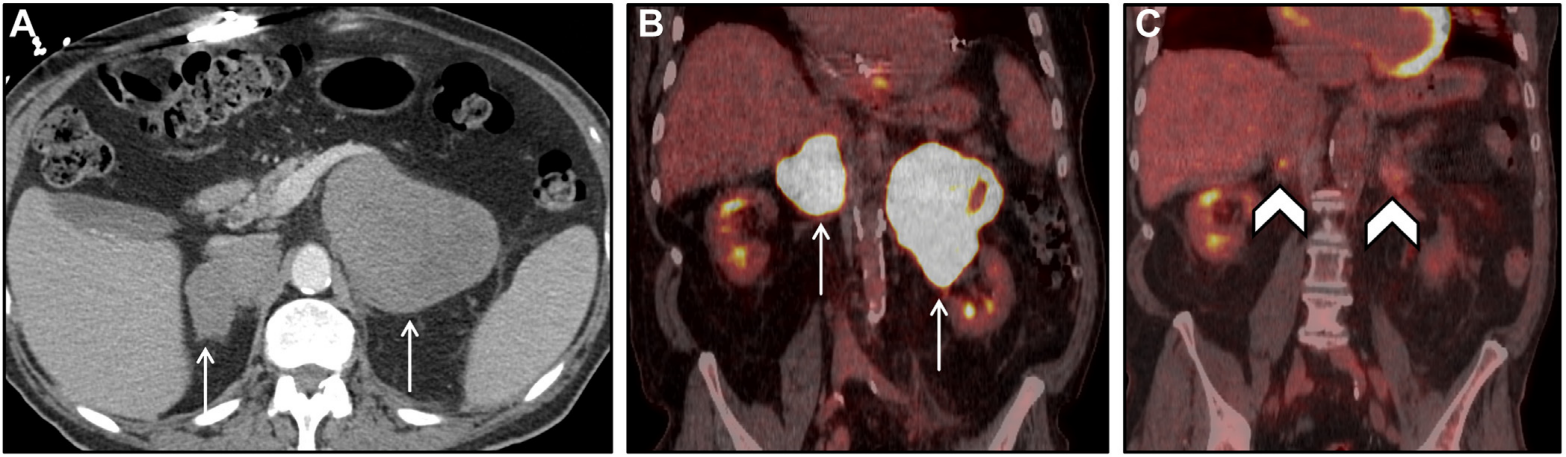
Bilateral adrenal lymphoma. (*A*) Axial CT image with contrast demonstrates large bilateral adrenal masses in a patient with diffuse large B cell lymphoma. (*B*) Fused coronal FDG PET/CT image demonstrates marked FDG uptake. (*C*) Follow-up fused coronal PET/CT image after treatment demonstrates interval decrease in size and FDG uptake of the bilateral lesions (arrow heads) in keeping with favorable treatment response. *CT* = computed tomography; *FDG PET* = fluorodeoxyglucose positron emission tomography.

Primary malignancies of the adrenal gland commonly originate from the adrenal cortex or medulla; rarely however, patients can also develop primary adrenal lymphoma. This is a 76-year-old male with end-stage ischemic cardiomyopathy and atrial fibrillation requiring automatic implantable cardioverter defibrillator (AICD) and left ventricular assist device placement. He developed purulent drainage and induration at his AICD pocket and flank pain. CT abdomen/pelvis was obtained to characterize flank pain, initially thought to be due to a distal abscess caused by hematogenous spread from infection at AICD site. CT instead demonstrated new large bilateral adrenal masses (the largest measuring up to 10 cm in diameter); patient had normal adrenal glands on CT obtained 1 year prior. FDG-PET obtained days after this CT demonstrated AICD-associated infection and enlarging hypermetabolic adrenal masses concerning for malignancy. Retroperitoneal mass biopsy demonstrated diffuse large B-cell lymphoma. Patient received combination cytotoxic chemotherapy with rituximab and prednisone with good partial response to therapy (Fig. 10).

## Discussion

With the widespread prevalence of cross-sectional imaging, unilateral and bilateral adrenal masses are increasingly achieving diagnostic recognition. Here, we present a visual case series of bilateral adrenal masses to illustrate the spectrum of these lesions and highlight the varied clinical contexts in which they occur. All patients with adrenal masses should undergo hormonal evaluation; this is especially true for bilateral lesions which may be associated with both hormone excess and deficiency. Bilateral masses are also more likely to be associated with malignancy and tumor susceptibility syndromes.^2^ An understanding of the hallmark radiographic characteristics in different disease contexts (Table) can aid in accurate diagnosis and timely intervention,^8^^,^^9^ particularly for patients with rare adrenal diseases.

**Table:** Typical Features to Distinguish Common Adrenal Masses

	ACA	Adrenocortical hyperplasia	ACC	Metastasis	Pheochromocytoma	Myelolipoma	Hemorrhage
Presenting clinical features	Incidentaloma; evidence of hormone excess	Hormone excess; in rare cases, hormone deficiency	Incidentaloma; suspected or known susceptibility syndrome; hormone excess; mass effect	History of or suspicion for malignancy; if bilateral, new adrenal insufficiency	Hormone excess; suspected or known genetic syndrome	Incidentaloma; suspected or known CAH; occasionally, mass effect	Trauma, sepsis, disseminated intravascular coagulation; acute onset flank pain; new adrenal insufficiency
Often bilateral?	No	Yes	No	Yes	No; can be bilateral in syndromic cases	No; can be bilateral in CAH	Yes
Size	Small (< 4 cm)	Uniform cortical enlargement, sometimes with nodularity	Variable; typically large (> 4 cm)	Variable; often small, multifocal	Variable; typically large (>3 cm) if hormone excess present	Variable	Variable
Texture	Homogeneous	Homogeneous	Heterogeneous; can have necrosis, hemorrhage, calcifications. May invade adjacent vessels with tumor ± bland thrombus	Heterogeneous; can have hemorrhage, calcifications, necrosis, cystic components	Heterogeneous; can have cystic and hemorrhagic components	Heterogeneous; can have myeloid areas, lipomatous areas, and calcifications	Heterogeneous with areas of hemorrhage, can also have cystic components, surrounding fat stranding depending on etiology. Calcifications may develop once chronic
Shape	Round	Adreniform, sometimes with nodularity	Irregular, may invade adjacent structures	Variable	Round	Round	Variable
Unenhanced CT attenuation	<10 HU; cortisol-secreting may be < 20 HU	<10 HU	Typically > 20 HU and heterogeneous. Presence of low density areas does not exclude ACC as it might contain microscopic fat	Typically >20 HU	Typically >20 HU	Variable within mass (lipomatous areas < -30 HU)	Depends on acuity; often high attenuating initially (eg > 50 HU) and liquefies to low density over time
CT contrast enhancing?	Homogeneous enhancement with rapid washout	No	Yes, often heterogeneously	Yes	Yes, avidly	Myeloid areas can enhance	No
MRI intensity (reference liver)	Isointense on T2; cortisol-secreting may be hyperintense on T2	Isointense on T2	Mildly hyperintense on T2, often heterogeneous; areas of necrosis can be T2-bright	Variable but often mildly hyperintense on T2; hypointense on T1 if no hemorrhage	Markedly hyperintense on T2 (lightbulb sign).	Depends on content; fat is hyperintense on T1 and on T2	Variable depending on acuity
FDG-PET avid?	Variable; often not avid	Variable	Heterogeneously avid	Variable, typically avid	Variable, typically avid	No	Variable

Abbreviations: ACA = adrenocortical adenoma; ACC = adrenocortical carcinoma; BMAH = bilateral macronodular adrenal hyperplasia; CAH = congenital adrenal hyperplasia; CT = computed tomography; HU = Hounsfield units; FDG-PET = fluorodeoxyglucose positron emission tomography; MRI = magnetic resonance imaging.

## Disclosure

The authors have no conflicts of interest to disclose. Given their role as Editor, Dr Sina Jasim had no involvement in the peer-review of this article and has no access to information regarding its peer-review. Full responsibility for the editorial process for this article was delegated to Dr Mira Sofia Torres.
